# Distributing Summer Meals during a Pandemic: Challenges and Innovations

**DOI:** 10.3390/ijerph19063167

**Published:** 2022-03-08

**Authors:** Brooke L. Bennett, Kim M. Gans, Kara Burkholder, Julia Esposito, Sarah Wen Warykas, Marlene B. Schwartz

**Affiliations:** 1 Rudd Center for Food Policy & Health, University of Connecticut, Hartford, CT 06103, USA; kara.burkholder@uconn.edu (K.B.); julia.s.esposito@uconn.edu (J.E.); sarah.warykas@uconn.edu (S.W.W.); marlene.schwartz@uconn.edu (M.B.S.); 2 Department of Human Development and Family Sciences, University of Connecticut, Storrs, CT 06269, USA; kim.gans@uconn.edu

**Keywords:** summer meal program, school meals, school nutrition programs, school food services, COVID-19 pandemic, food service directors

## Abstract

The USDA summer food programs provide meals for children when school is not in session. Although the COVID-19 pandemic has created challenges for food distribution programs, many regulations have been waived, providing opportunities for new approaches to meal distribution. The aim of this study was to identify practices designed to increase program participation during the summer of 2021. Semi-structured interviews were conducted with food service directors (N = 16) in a northeastern state. Questions addressed meal distribution methods; perceptions about facilitators and barriers to family participation; communication strategies used to reach families; and engagement with community partners. The responses were analyzed using an immersion-crystallization approach and four themes emerged: new opportunities for innovation due to the waivers; the importance of collaboration with community partners to increase reach; ongoing logistical challenges due to the pandemic; and the challenge and importance of reducing the stigma of participation. These findings underscore how the USDA waivers increased food service directors’ ability to flexibly and creatively solve problems related to summer meal delivery. The FSDs believed that several of the waivers helped them increase participation in the summer meal program, suggesting that permanent changes to the summer meal regulations may be appropriate.

## 1. Introduction

Approximately 13 million children in the United States live in a food-insecure household [1]. During the school year, many families rely on the U.S. Department of Agriculture (USDA) school meal programs (e.g., School Breakfast Program and National School Lunch Program) to provide nutritious meals for their children. In the summer, however, these households lose access to school meals and their rates of food insecurity increase [2]. The purpose of the USDA’s summer meal programs is to fill in this gap. There are two federally funded summer meal program options: the Seamless Summer Option (SSO) of the National School Lunch Program, which allows school food authorities in districts with free/reduced rates of 50% or higher to continue serving meals during the summer, and the Summer Food Service Program (SFSP), which has different nutrition standards than the National School Lunch Program (NSLP) and can be sponsored by schools or other organizations that serve youth [3].

The summer meal program reaches significantly fewer children than the meals served during the school year. In 2019, approximately 22 million children relied on the National School Lunch Program for meals during the school year; in contrast, less than 4 million received meals during the summer [4]. One barrier that is removed in the summer is the requirement to qualify for free meals; during the summer, all meals are served at no cost to children 18 and under, regardless of family income level. There is evidence that summer meals can alleviate the increase in food insecurity among households with school-aged children. In one study, Nord and Romig examined the seasonal differences in the rates of food insecurity among households with and without school-aged children. As predicted, they found that seasonal differences in food insecurity among households with school-aged children were greater in states with smaller numbers of Summer Food Service Program meals and summertime school lunches [2].

The COVID-19 pandemic brought unprecedented challenges to school meal programs across the United States, including those in place during the academic year and during the summers of 2020 and 2021. In response to the spike in food insecurity in the early pandemic [1], the closure of school buildings and the onset of remote instruction, the USDA offered several important waivers to meal program regulations. First, districts with free/reduced rates lower than 50% were allowed to participate in the summer meal programs (the SSO and the SFSP). There were also waivers that allowed food service directors to deviate from many of the previously required practices. These included permission to serve (a) grab-and-go meals; (b) meals that did not fully meet the school meal pattern requirements; (c) multiple meals at one time; and (d) children’s meals to parents or guardians when the child was not physically present [5,6]. Several studies have documented how the pandemic impacted school meals during the academic year, and in particular how the USDA waivers allowed flexibility and innovation [5,7,8]. These waivers also applied to summer meals, providing food service directors additional flexibility in how to structure their programs.

Food service directors (FSDs) play a key role in the establishment and operation of the summer meal program. In the summer meal program, FSDs are responsible for implementing the program and making almost all strategic decisions that will impact participation and nutrition. For example, food service directors select the site locations, pick and design the outreach methods, and decide the type of meals provided to participants. Therefore, it is crucial to understand what these directors perceive as strengths and challenges of their programs. It is also important to gather insights from FSDs on a large scale so that programs can learn and benefit from each other. In this study, we aimed to explore FSDs’ perspectives on what works for them, how they make decisions, how COVID-19 plays a role, and where they see the program going during this time of tremendous change.

The aim of this study was to identify pandemic-related challenges and new practices designed to increase participation in USDA meal programs during the summer of 2021. Specifically, we aimed to evaluate food service directors’ perspectives related to family participation, communication methods, food quality, and collaboration with other local food services. We hypothesized that the federal regulatory changes to the summer meal program would significantly impact the structure and delivery of meal programs on a local level. We also hypothesized that food service directors would report pandemic-related barriers such as staff shortages.

## 2. Materials and Methods

This study employed a phenomenological qualitative approach. We conducted key informant interviews with district food service directors across Connecticut from June to July 2021. This study was deemed exempt from full review by the University of Connecticut institutional review board (Exemption#X21-0116).

### 2.1. Setting

Notably, Connecticut has large economic disparities [9] which are reflected in the free or reduced-price eligibility of school districts across the state; there are 11 large urban districts where more than two-thirds of the students are eligible for free or reduced-price meals and approximately two dozen districts where fewer than 15% of students are eligible. In summer 2019, the summer meal programs in Connecticut (CT) distributed meals to over 37,000 children aged 18 years and younger at 656 locations statewide [10]. Due to the waivers released in 2020 by the USDA in response to COVID-19, some school districts were eligible to participate in the summer meal programs for the first time. FSDs from these districts were required to submit a proposal to the Connecticut State Department of Education explaining how a summer meal program would benefit children in their communities, particularly those from low-income households or those who were economically impacted by COVID-19.

For the present study, the research team selected FSD interviewees to maximize the demographic diversity of the school districts represented. There are nine district reference groups (DRG) in Connecticut. Each DRG reflects distinct characteristics including education level, urbanicity, and town size. For example, DRG E is categorized as very small and rural with the lowest enrollment rates (i.e., Montville, Thompson) while DRG I is made up of the state’s largest cities and urban centers with highest enrollment (i.e., Hartford, New Haven).

In the summer of 2021 in Connecticut, there were no lockdowns or pandemic-related activity restrictions. During the study period (June and July 2021), mask mandates were dependent on individual city ordinances [11]. Additionally, as of 1 July 2021, 60.8% of citizens in Connecticut were fully vaccinated and 67.1% had received at least one dose [12].

### 2.2. Key Informant Interviews

Twenty-three FSDs across all nine DRGs were invited to participate in this study via email. The purpose of this study was explained in the body of the email invitation and reiterated at the beginning of the interview. Everyone was informed that this study was sponsored by the Connecticut State Department of Education. Four FSDs never responded to the invitation and three declined to participate. The final response rate was 70% (16 FSDs) and eight of the nine DRGs were represented. The missing DRG (DRG A) is characterized by high income and education rates. These school districts do not typically have summer meal programs because they are ineligible based on free/reduced rates during the school year. The final sample included 3 districts with a free/reduced rate less than 25%, 5 districts with a free/reduced rate between 25 and 50%, and 8 districts with a free/reduced rate greater than 50%. The final sample consisted of five male and eleven female food service directors. Of note, all sixteen FSDs also run their school lunch programs during the academic year. Additionally, all food service directors are required to complete 12 hours of annual training in nutrition per the USDA [13]. Participating FSDs were offered $500 that could be spent on materials to promote their summer meal programs. The most popular items selected were staff t-shirts, insulated food bags, and magnets with a QR code that links to the district summer meal website.

We conducted individual, 30 min, semi-structured interviews with sixteen FSDs, each from a different school district, via a videoconferencing platform (WebEx, version 41.6, Cisco, San Jose, CA, USA). The first five interviews were led by a senior researcher with experience conducting interviews (M.B.S.) and included a team of undergraduate and graduate student research interns. The remaining interviews were conducted by the team of student interns. The question guide was created for this study by the research team and in consultation with the Department of Education. We asked open-ended questions about meal distribution methods, factors influencing site location, barriers and facilitators to participation, student participation and reach, communication methods used to increase participation, work with community partners, and long-term future needs. The questions used in the interviews are listed in Appendix A. Interviews were audio-recorded and transcribed. The online meeting platform used for the interviews (WebEx) provided an auto-generated transcript. A member of the research team who had participated in each interview reviewed the transcripts and made corrections to the auto-generated transcripts where appropriate. After the 16th interview, the team determined that data saturation had been reached.

### 2.3. Qualitative Data Analysis

Key informant interview transcripts were analyzed using the immersion-crystallization approach [14]. During the immersion process, two doctoral-level researchers (M.B.S. and B.L.B.) conducted in-depth reviews of the interview transcripts while taking detailed notes to identify key aspects of participation in the summer meal program. One graduate student researcher (S.W.W.) and two undergraduate researchers (K.B. and J.E.) developed an initial set of codes based on patterns identified in five interviews and met with a fourth researcher (K.M.G.) for peer debriefing. Based on this meeting, the team established a coding guide. We analyzed the remaining interviews and added additional codes as additional patterns were identified. Three researchers (K.B., S.W.W., and J.E.) coded the interviews using Dedoose (version 9.0.46, Dedoose, Los Angeles, CA, USA), a qualitative research software and selected quotes exemplifying the codes. Each transcript was double coded. After coding was complete, the team reached consensus on the themes from the interviews. The final codebook was approved by the full research team.

## 3. Results

Four main themes emerged from the analysis: innovation, collaboration, challenges, and overcoming stigma. Details on each theme and relevant subthemes follow.

### 3.1. Innovation

#### 3.1.1. Innovation Due to Waivers

Participating FSDs reported that the USDA pandemic waivers allowed for flexibility and creativity. This was most easily seen in the reported changes to meal distribution methods. The FSDs reported a wide range of distribution methods including options such as drive-up, a walk-up window, and grab-and-go meals. FSDs reported that the non-congregate waivers allowed them to distribute multiple meals at once. Some FSDs reported that they distributed breakfast and lunch at the same time, while others used the waiver to give out “weekend packages”, which contained hot meals for Friday and cold or frozen meals for Saturday and Sunday. Similarly, some districts reported giving out 7 days’ worth of food on one day and others reported doing “holiday packages” to cover missed days such as 4 July. FSDs highlighted the benefits of this type of meal distribution. First, they found that this method of distribution meant no food waste for their programs. Some directors also pointed out that the waivers meant that children no longer must throw away food that they do not consume on site. Other directors pointed out that this method of distribution also meant fewer hours for staff to cover, and less electricity used in the kitchen, thereby saving the district money.

Some FSDs used the waivers as an opportunity to innovate further and find ways to bring the meals to the community. A few districts packed vehicles with coolers and stopped at sites such as apartment buildings, parks, and other places that families/children might be. These directors described the creative efforts they undertook to do this safely including converting the meals to cold meals such as deli sandwiches and hummus, typing out reheating instructions for the frozen meals, and reaching out to community partners for help getting coolers, ice bags, and other needed items. One FSD explained that they were motivated to go mobile because transportation becomes an “artificial barrier” once organizations start transporting meals or delivering meals to homes. Two districts embodied this idea and used bus routes to maximize their chances of reaching the public. One described working with the transportation department in their town to plot out the route to ensure that the furthest anyone would have to walk was 10 minutes. She said:

*The main way that we distribute meals to anyone in the public this year is through bus routes. So, we pre-bag 7 days’ worth of food into big brown paper bags and we go out once a week on to, oh gosh what is it? 30? It might be, like, 35 or so different bus stops, so each stop runs for about 10 minutes. People show up at the designated time, we give them the bag with all the food in it and the milk and everything, and then they have a week’s worth of food.*

FSDs also highlighted that the waivers allowed parents to pick up meals for multiple children, which increased participation rates. Some districts even partnered with other community organizations to cover adult meals as well. For example, one FSD said, “adult meals are a really big thing. We actually we got a grant from [local non-profit] a couple of years ago, and we did adult meals in some of our parks.” One director estimated that the number of meals distributed increased from 200 to between 400 and 500 due to parents being able to pick up meals for all their children. The FSDs discussed the importance of thinking about what the parents need, and then meeting those needs in order to serve the children. For example, one district described having an extended meal service from 3:00 to 6:00 p.m. to better accommodate parental work schedules. 

FSDs also described combining the school meal distribution with other food programs in order to maximize the benefit for families of making the trip. For example, the Department of Defense commodity food funding first extended into the summer in 2017 [15]. Multiple FSDs reported they were able to use this funding alongside USDA waivers which allowed for distribution of produce in non-congregate settings [16] to increase the amount of fresh produce they could distribute. One district used the DoD funding to create a distinct “farm stand” at their summer meal site, and another district gave out additional “Farm and Family Boxes” full of produce at the same time as the summer meals. Both districts reported seeing an increase in participation after they advertised the availability of the additional produce on social media. 

Overall, FSDs expressed their hopes that the waivers remain in place following the pandemic and their concern if they do not. One FSD said:

*The difference between being able to give people meals and not have them consume them on site, is humongous. When we go back to having to have people consume food on site, I know that not many people will be doing that because they can’t sometimes.*

#### 3.1.2. Increasing Participation 

Other examples of innovation and creativity emerged during discussions of strategies the directors use to increase participation. Two districts collected data through parent surveys in order to gauge interest in the program and obtain an understanding of what types of meals families preferred (heat and go, sandwiches, etc.). Other districts reported using unique “add-on” programming to increase participation. For example, one district reported that they had started a school garden with a local non-profit, both so that the children could work in the garden when they came to pick up meals and so the school could offer the produce grown there to families. Another district reported offering homework help as an incentive: “If you need help with your math packet for free, come to this site and we will have lunch and will do part of your math packet with you”. Lastly, one district reported using a theme or a brand to tie together programming across the summer:

*I have done planting zucchini seeds because one of the Put Local on Your Tray’s [a local organization] themes was about zucchini and squash. I had a table set up and the kids get to come, and I talk about the zucchini, and I show them what a zucchini is. Then I would put seeds in plastic zip lock baggies, and I told the kids that this is what they put in their little greenhouse at home and when it grows bigger, you can take it and put in a pot. I then showed them - this is a whole zucchini - and I let them try it raw, and then I brought some that was cooked for them to try. I then gave their parent one zucchini to take home with them so that they can cook it at home that night, with the recipe on how to cook it, something simple. And we use local CT farmers to get those products.*

### 3.2. Collaboration

FSDs reported that they used collaborations to facilitate outreach and bolster participant engagement in the summer meal program. Firstly, FSDs reported collaborating with other school districts. Some reported reaching out to neighboring towns that were not doing a summer meal program to make sure they knew those families were welcome across the district line and others reported promoting nearby districts that also had sites to make sure families could find a site that fit their schedule and transportation needs. Secondly, FSDs described collaborating with local non-profits and community partners in a variety of capacities. For example, multiple districts reported working with community partners to pick the location of summer meal sites, such as the town government’s Parks and Recreation department or local Community Cultural Centers that were familiar with the neighborhoods in the district and knew where would be the most need. Other directors reported that they consulted with individual community advocates who were familiar with their district demographics to help guide site locations. Other districts engaged community groups to ensure that people knew about the availability of summer meals; the non-profit groups mentioned included soup kitchens, local food pantries, Rotary and Lions Clubs, Councils of Churches, local dairy councils, university extension programs, and Community Action Agencies. The directors described these relationships as “mutually beneficial” because all of these organizations are attempting to reach the same demographic of families. In some cases, the relationship was mutually beneficial because the FSDs were able to prevent food waste by donating the leftovers to other local food distribution programs. It was notable that some FSDs reported that they were able to connect with other groups to help with outreach because they served as leaders in community organizations and boards outside of the school district. 

Several districts reported collaborating with local retailers to boost outreach and capacity. For example, one district asked a local drugstore and a local fishing and hunting retailer for donations to hold seasonal raffles for summer meal participants. Another FSD asked for the donation of time by asking food service brokers (the salespeople that connect branded products to schools) to come work a few shifts at a summer meal site to better understand the people they are serving with their products. FSDs also discussed collaborations with their local government and city leaders. For example, some districts worked with fire departments to have them bring a fire truck to school during meals, while others worked with the police to facilitate events such as “pizza with the police”. One district coordinated with the library book truck so children could pick up books and meals at the same time. Another district was able to coordinate help with staffing from their town’s Office of Community Services. Another district worked directly with their town hall and emphasized how the pandemic had shifted the local government’s thinking about how to serve children:

*The approach was, “We feed students in schools”. Then over this last year, we migrated to … “We serve children, we feed children in the city.” Once you take the approach that you feed children in the city, [you’re] no longer just working with school officials.*

#### Outreach and Communication

FSDs reported using collaborations to facilitate and improve their outreach efforts. Notably, multiple districts reported collaborating with a state-wide anti-hunger advocacy organization called End Hunger Connecticut! (EHC!) to promote their summer meal sites. They reported receiving promotional materials such as flyers and lawn signs from EHC! and relying on EHC! to distribute the materials in their towns. Other districts reported similar relationships with organizations such as the local community’s Hunger Action Team and their local United Way affiliate. Directors also reported using spokespeople within their own school district to help communicate with parents; for example, having the district superintendent or one of the school principals send out ParentSquare (a communication tool that serves as an interface for families and staff) messaging instead of the nutrition department. One FSD said: “most impactful (marketing tactic) was definitely the Superintendent’s ParentSquare message because it came from him and if it came from me, because I can ParentSquare too, but I am the director of nutrition services and no one reads my emails. But if it came from the Superintendent, people actually pay attention to it”.

In addition to traditional marketing to parents through emails, texts, posts on the district website, and robo-calls, many districts reported trying new or innovative approaches to get people’s attention. For example, some districts made press releases which they sent to the local paper and radio stations with the hopes of a free public service announcement. One district was successful in getting their local paper to do a featured piece on the farm stand they had set up to accompany the summer meals at their distribution site. Other districts reached out to community leaders, including state representatives, social workers, and church leaders, to help spread the word. Some FSDs reported using electronic message boards such as at the local fire department and high school to advertise. Others elected for more targeted approaches such as advertising through the phone bank for English language learners: “we have an English language learners group that does phone calls and so they set up a phone bank and they call house by house, you know, to all the folks in the English language learners organization”.

FSDs reported a mixture of in-person, digital, and print outreach approaches. Some districts tried new strategies including taking out print ads in the town newspaper and placing an ad-wrap on a box truck. Other districts found success by hosting promotional events at the beginning of the summer. For example, one district used grant money to hold a “party in the park” barbeque to get people out for a free meal and promote the summer meal program. Social media was commonly cited as one of the most effective marketing strategies. Multiple directors reported that they needed to be strategic with their posts in order to drive up engagement on social media sites such as Facebook. Some of the lessons they learned were: people like seeing a picture of the menu for the week; people react more to posts that include pictures; and people like seeing the actual food. One FSD shared pictures of the produce available at the farm stand they set up at their summer meal distribution site. Another person remarked on a particularly successful social media post that featured food:

*I took a picture of a 7-day bag. We took everything out, laid it out on the table, and put it in the front of a sign and took a picture of it and said, “This is what a 7-day bag looks like”. That seemed to be pretty popular.*

### 3.3. Challenges 

FSDs noted how overseeing the summer meal programs in 2019, 2020, and 2021 was like running three different programs. When asked about their experiences during the summer of 2021, FSDs highlighted a series of continued logistical difficulties linked to running a program mid-pandemic; new challenges for families that were not an issue in 2020; and the unique challenges related to running summer meal programs in higher-income towns.

#### 3.3.1. Logistical Challenges

FSDs reported a series of challenges and relevant considerations related to logistical concerns. For example, they highlighted that each needed to be approved by both the Connecticut State Department of Education (SDE) and the town. Setting up grab-and-go sites required an analysis of traffic patterns (in case there were long lines of cars); construction projects at school sites; and the geography of the neighborhood when asking people to walk. For example, one FSD said: “we want to make sure that we go up into the hills, [the town] is very hilly, on the other side of town and set up a site there”. Directors reported that they needed to consider the safety of the site in terms of possible violence in the neighborhood or whether students would need to cross a busy street to get to the site. Selecting the locations for site relies on the directors and staff knowing their town well; for example, one FSD stated, “It’s really more about trying to get to the communities and the neighborhoods where we felt people wouldn’t be able to get to us otherwise”.

The FSDs also highlighted a series of equipment and supply considerations related to operating a summer meal site, such as the need for coolers and ice packs to keep meals at the proper temperature. FSDs reported that they also must ensure that they have adequate kitchen space as well as refrigerator and freezer space when preparing meals. Access to the foods they need was also sometimes an issue, as one FSD noted:

*During the summer a lot of our distributors don’t have all the same products that you’re used to getting during the school year. So, you don’t have as much access to somebody in the supplies that you normally would serve.*

The FSDs described difficulties in selecting hours at each site because of the conflicting needs of parents versus staff. They knew that parents were more likely to be able to pick up meals in the evenings; however, it is more expensive to staff evening hours because the union requires overtime pay. Similar concerns were cited for weekend and holiday hours. FSDs also reported that challenges around outreach efforts were a lack of time, money, and staff. Notably, FSDs commented on how staff are feeling burnt out after a year of managing meals in hybrid schools. For example, one FSD said:

*Our program, we haven’t stopped feeding children. It seems like we took a week off last week when school closed. I think some of my staff are just tired and they’re burnt out. Okay, because we’ve been on the front line since this thing started, we opened on March 16th, and we haven’t closed until last week. We get a couple of days in between, so I think they’re just tired. They need a break.*

Lastly, they described the challenge of predicting participation each week, noting that it can be influenced by the outside temperature, rain, and proximity to food stamp distribution date. For example, one FSD said: “Sadly, I know today’s numbers are going to dip, not only because of the heat, but because it’s the 1st of July. Once peoples’ food stamps come out, they stop coming. I don’t know how people can’t understand that you could buy more with your food stamps if you come to our meal sites as well”.

#### 3.3.2. Challenges of the Pandemic 

FSDs reported a series of challenges unique to the 2021 summer as the COVID-19 pandemic and its impact on summer meals continued to evolve. The FSDs underscored the need for constant flexibility in approaching the ever-changing situation. For example, they reported that it was particularly difficult to estimate the number of meals they needed to prepare each week. Some reported that participation started out slow but increased as the summer went on. Other directors reported that the estimates made based on 2020’s numbers were way too high, resulting in too much food ordered or staff costs that did not match the number of people showing up. The directors attributed this change in participation to a few potential causes: (1) children were back at camps that were closed in 2020, so they received food through those, (2) families were back at work but still in need, (3) people were no longer working from home, so they could not swing by on their lunch break. The FSDs also explained that grant opportunities, such as those through the American Recovery Act, led to additional programming for kids that contributed to difficulty in scheduling meals for each of the programs in the district. One participant explained,

*And so everyone applied for all of these little grant programs. So, they could have 2 weeks of a boot camp or 2 weeks of extra enrichment, or 2 weeks of “get the kids out of the house and have fun because they’ve been cooped up for a year” kind of a thing. And so they had all these little groups have all these little programs, but they’re only running for 2 weeks or 14 days here and 3 days on the other end, or we’re here for 3 weeks. So, none of them are like, long programs that normally run the gamut of my summer.*

FSDs reported that operating for another summer in the pandemic also led to some unique staffing issues. For example, they reported that they were having a hard time staffing because people were burnt-out from the additional stress. Some directors also noted that their staff were having trouble getting daycare for their own children with unexpected shutdowns or coronavirus exposures. The FSDs reported that these challenges influenced site placement and hours. For example, one director was unable to get the needed security staff for one of their sites and found that families were struggling to get into the building as a result. Directors also reported that it was difficult to get staff to work when they were not able to offer a competitive wage.

On the other hand, some directors discussed their efforts to boost morale by trying to increase staff buy-in to the cause and improve organizational culture. Some districts reported they had success in providing staff incentives from community partners. For example, one FSD said:

*Another [local shoe store] had a bunch of products that were just not moving, sitting on their shelves. It was like 140 pairs of sneakers and I was able to get that to come here through the approval of the superintendent. Every staff was able to come in and get their size shoe for themselves, or for their kid. So those little things. I don’t have the access to give raises. But little things that we could do to show appreciation goes a very, very long way.*

#### 3.3.3. Barriers for Families 

The FSDs reported that participation numbers were hampered by a series of barriers that families faced, including logistical concerns, such as a lack of transportation; short windows of time when meals can be picked-up; inconvenient hours for pick-up; and single parents who may not have time or childcare so they can run out and get the meals. The directors also reported that lack of participation could be due to lack of awareness of the program, or a misunderstanding of how the summer meal program operates. They explained that some families did not know about summer meals or were confused by rules since they are different than during the school year. Some directors reported that concerns about COVID-19 transmission could have been a barrier and reported that they attempted to improve communication about how the food was packaged and asked their staff to continue wearing masks. 

Other directors reported that the menus were likely contributing to lack of participation. They explained that people prefer when there is variety, and variety was more limited in the summer meal program than the regular school year. FSDs also reported that families prefer to see the menu in advance; however, one noted that they were reluctant to post menus because they were worried they would run out of the posted meal. Other directors reported they have received more requests for vegetarian and vegan items in recent years. 

One of the most cited barriers across interviews was stigma or embarrassment. One director reported that they stopped asking people to sign up in advance once they realized that concerns about privacy were dissuading people from participating. Other directors reported that they heard from families that they do not want to participate because they did not want to take food from a family that might need it. One director noted: 

A lot of people say, like, “I don’t need it. I don’t want to take it away from someone who does” and we always say, you know, “there’s plenty of food to go around. This is really supporting our program, it’s supporting our program to serve better food, it’s supporting the employees who work for this program. It’s, it’s ultimately supporting the local economy of the city”.

Another director reported that political beliefs likely played a role in the stigma around using the summer meal program:

*I know that there are people that need it and there are definitely people in my town that I know that are conservative… who don’t believe in taking handouts from the government and that’s the way they see it.*

#### 3.3.4. Unique Challenges in Higher-Income Towns 

As noted above, due to changes in eligibility due to the ongoing COVID-19 pandemic, some districts were newly eligible to participate in the summer meal program. As a result, the directors reported some challenges associated with operating a meal site in a higher-income town. For example, some FSDs were surprised by the need in their communities:

*I was surprised by the need in our community and we are a pretty low free and reduced district. But, my take of this is that don’t judge a book by its cover because there is a greater need than we know and it’s important to let people know that help is available.*

Another was focused on how they could sustain the program in future years:

*I won’t be getting enough people in just on the meals alone, I need to have something that draws people in, like the farmer’s market and I would need some funding for that. But it is vital in these communities, even though it is vital for a very small population and even I had no idea, how much it meant for these families.*

FSDs also reported concerns about the families who need the summer meal program and their lack of ability to get other services in their district. For example, one director said:

*But even if there wasn’t a pandemic, people assume that there is not a great need in communities like mine, but in some ways, there is a bigger need because there are no other services for these families. Because in [lower SES city], you can walk anywhere and get the services you need, but here, if you are a family in need, you are screwed, because there is nothing there for you because we are a tiny rural community and for those families (very small amount), the need is greater.*

FSDs also highlighted that the students who were eligible for free or reduced-price lunches might experience even greater stigma in higher-income districts due to the discrepancy in family income. One director said:

*When if you are the poor kid in town, you are really in the minority. So doing things like the farm stand or the cheeseburger grilling day or just having the idea that this is our community site, this is a place where families see each other in line and get out of their cars and talk to each other and that is incredibly important to getting rid of the stigma and finding ways to get this program known.*

Lastly, directors expressed concern about the lack of outreach in these higher-income areas and how it may have made it difficult for families in need to find meal sites in neighboring districts. One FSD said:

*Not, even just community to community because there are areas, like we mentioned, where they don’t qualify but there are still people there that could use help and if they know that they can go to the town next door, they could get help. We work during the school year in [names of towns], but we also work in region 7 which is a fairly wealthy district, but in there, there are pockets of people that need help. We deal with large variations in socioeconomics. We try to put the information out there for everybody as we know that there are a lot of people that need help.*

### 3.4. Overcoming Stigma

In addition to discussing stigma as a barrier to participation, the directors also discussed trying to decrease stigma by focusing on why families use the program. 

#### 3.4.1. Why People Use the Program

FSDs reported that the main driver of program use was the economic benefit to the family. They also attributed higher rates of participation to logistical factors including flexibility in pick-up time, open sites in easy-to-access areas around bus lines, and sites in areas where kids already congregated such as a city park or pool. The FSDs also reported that families were incentivized to participate when they liked the menu and explained that children like hot meals and FSDs see upticks in participation for particular meals such as pizza or Jamaican beef patties. One FSD said:

*Knowing what the kids really like is important and making sure that you drive a menu that they want. I found that out a lot during remote meals on days where I just do a turkey sandwich it didn’t really bring the crowd, but if I did a Jamaican beef patty, I had a lot I had cars lined up with kids. It’s just fun. So I learned really fast what they liked what they didn’t like, what they’re willing to come out for. So, I think that’s important.*

Lastly, FSDs perceived that some families may have used the program because they wanted to help the school and ensure the program does not get eliminated in the future. 

#### 3.4.2. Combatting Stigma

FSDs reported that they put effort into emphasizing the components of the program they thought would attract families of all socioeconomic levels in order to reduce the amount of stigma felt by people participating. For example, one district found that their use of a farm stand was successful in attracting new participants to the meal sites. Other districts held events that emphasized fun. For example, one district had the principals and vice principals grill for the children on National Grilling Day. In addition, some districts felt that the flexibility in distribution methods could help combat stigma. In particular, they cited that drive-ups for grab-and-go meals were appealing:

*It seems to be more of a sustainable program because the parents don’t have to be embarrassed because I think that if you made them park their car, come into the school, say “I’m here for free meals” you might lose a lot of people, but when they can just pull up and say “2 kids” and pop their trunk…*

Some FSDs reported that they made a deliberate effort to combat stigmatizing attitudes within their staff. Some reported that they spent time emphasizing to the staff that they were part of the community and worked to enhance staff buy-in to the mission. Other districts held staff trainings to combat stigma. After a staff member expressed concern about not knowing when someone is taking advantage of the program, one director said, “And, we have to do our due diligence here, but we cannot control what everybody else does. And that doesn’t mean that we should make the other 99% of people feel uncomfortable about coming to us, right?” 

FSDs reported explicitly working to combat stigma in their marketing and by using community influencers. First, in their messaging, directors reported feeling the need to “brand” themselves. They worked to create a professional, coherent appearance for the program by using logo tents, vans with wrapped ads, and uniforms for employees. In the messaging itself, they reported that they worked to de-emphasize that the meals are for people who need them and instead used messages such as “5 reasons to try school meals” where the reasons included (1) meals at no costs to families, (2) eating healthy and nutritious, (3) available to all children aged 18 and younger, (4) local farms and businesses, and (5) support your school district. Others reported that they used messages to appeal to all families, such as saying that these meals are “for moms that are just tired of making lunch” or pitching the program as a way to support your community and local farmer. 

Several directors also spoke about the importance of marketing the actual meals themselves. For example, one director said:

*We have definitely gotten feedback from parents saying, “I can’t believe that this is what the kids are getting, it is like gourmet”. And I always reply back that it is not “your school lunch anymore; this is a totally different school meal”.*

Others echoed this message and used social media to market their food. For example, one FSD said:

*I also take pictures when the staff are making the meals, like grilled chicken Caesar salad and posted it on the Facebook pages so that people can see. And our numbers increased that day because everybody saw it. And, today, our numbers will increase because it is pizza, and everybody knows that it is pizza day.*

In their efforts to rebrand the summer meal program and combat stigma, some FSDs described a creative use of community influencers. For example, one district had the governor’s press conference at the summer meal site and Board of Education volunteers at their events. Another made the effort to recruit the Parent Teacher Organization (PTO) president to come to the site and post about it online. The director said:

*We have gotten the PTO president to come out to our site and we took a picture of her getting the stuff and posting it on Facebook. …we are celebrating the summer meals. So, getting the influencers to use the program too, we have learned a huge lesson there.*

## 4. Discussion

Through qualitative interviews with FSDs, this study sought to capture the experiences of food service directors striving to increase participation in the summer meal program during the second summer of the pandemic. Specifically, we were interested in their perspectives related to family participation, communication methods, food quality, and collaboration with other local food services. This study adds to the literature on summer meal programs and provides new insights regarding innovations and challenges brought on by the ongoing coronavirus pandemic and the unique opportunity provided by the COVID-19 waivers of certain program regulations. 

Several findings in the current study were consistent with the existing literature. For example, FSDs reported their perception of the most common barriers to participation for families, which included transportation and lack of knowledge about the program. This was consistent with barriers reported by families before [17] and during the coronavirus pandemic [8]. Additionally, FSDs’ enthusiasm about the USDA waivers and the flexibility it provided for them to be more innovative in their distribution methods was consistent with research conducted during the school year [5,7]. 

There were also several new themes and ideas which emerged from the interviews. For example, some districts with higher socioeconomic status were eligible to participate for the first time due to a USDA waiver that allowed them to submit a proposal to their state agency explaining how a summer meal program would benefit children in their communities [6]. Interviews with FSDs from these districts introduced the new challenge of managing stigma in wealthier districts. The overall emphasis from FSDs on being more active in combatting stigma as a barrier to participation was also a novel finding. Because our study was conducted during the COVID-19 pandemic, it is difficult to say whether this finding was linked specifically to the pandemic, or if the efforts to combat stigma would have occurred regardless. Future research should examine efforts to combat stigma by food service directors following the pandemic to see whether their perspectives remain the same. 

Additionally, in this study, FSDs provided several innovative marketing and outreach approaches as well as ideas for innovative collaborations with city departments and community partners, such as Parks and Recreation, Food Pantries, and Transportation Departments. Further, this study provided the first documentation of the unique challenges of operating a summer meal program during a pandemic. Overall, these findings and the innovations reported by the FSDs could be beneficial to other school districts across the state and country facing similar challenges of running a summer meal program. 

The strengths of the present study should be considered alongside its limitations. First, our sample was moderate in size with 16 subjects. Future research could explore similar questions with a larger sample size to see if the findings are similar in a larger, more diverse sample. Although consistent themes emerged from the interviews we conducted, future work is needed to quantitively examine the efficacy of the strategies outlined by the FSDs. Future research should quantitatively assess the implementation, as well as the costs and benefits, of the strategies described. For example, future research should examine the efficacy of various outreach methods described by FSDs to see if they increase participation. Additionally, future work should check the perspectives of FSDs alongside objective data such as patterns of program usage and staffing needed. An additional limitation of the present study is geographic generalizability. While we tried to obtain a sample representative of the districts that participate in the summer meal program in Connecticut, the findings may not generalize to states with a different geographic or sociodemographic makeup. Future research should examine similar research questions in other areas of the country. Additionally, future research should examine the implementation of these strategies on a national level. In particular, future research should examine the efficacy of the strategies outlined to reduce stigma, both for families using the program and among staff preparing and serving meals. 

Further, while it is a strength of this study that it provides insights on the challenges facing FSDs running a summer meal program during the coronavirus pandemic, it also may limit the generalizability of the findings. For example, for the findings more clearly linked to the pandemic, such as use of the waivers, it is unclear whether these findings will remain relevant for FSDs when the coronavirus pandemic ends. For other findings, such as the innovative approaches to outreach and attempts to combat stigma, it is difficult to say whether these findings would have occurred if this study was conducted independent of the coronavirus pandemic. Future research should consider repeating this study when COVID-19 is over to gain a better understanding of which findings were uniquely tied to the pandemic. Finally, while FSDs are key stakeholders in the summer meal program, perspectives are needed from others within food service. Future research should explore the perspectives of other stakeholders such as staff, school administrators, members of the state department of education, and community partners to get a more well-rounded perspective. Further, both quantitative and qualitative perspectives from families and students are needed to better understand their level of satisfaction with existing programs as well as barriers that prevent them from participating. 

## 5. Conclusions

Overall, this study adds to the limited evidence about the factors that may contribute to participation in and access to the summer meal program. The findings revealed that FSDs have adapted during the pandemic to be flexible in their approach and planning. The participating FSDs saw the waivers as an opportunity for creativity and innovation and believed that the waivers helped them increase participation in the summer meal program. In fact, there are advocates and policy makers who are interested in extending the waivers as well [18]. This suggests that future research should investigate whether some of these waivers should become permanent changes to the summer meal programs. The FSDs also emphasized their belief that it is important to combat stigma and collaborate with community and city partners. Lastly, FSDs outlined the logistical difficulties and unique challenges of operating in a pandemic. 

## Data Availability

This publication has used qualitative data that in its full form could be identifiable. The researchers do not have permission to disclose the transcripts to any person other than those authorized for the research project.

## Appendix A. Interview

Introduction/Overview

1. Can you give us an overview of how your organization is currently distributing summer meals? How does this compare to 2020? 2019?
2. What methods do you use to distribute summer meals? (there may be different methods in different sites). Which do you think is most efficient? Has the greatest reach? Which is the most challenging?
3. How did you decide on the distribution methods you are using? How did staffing needs or capacity factor into your decisions?

Locations/accessibility considerations

4. Can you describe the decision-making process your organization went through when selecting distribution sites? What factors did you consider?

a. Prompts—For example, available staff, experience with prior summers, safety, walkability, equity, accessibility.

Barriers and Facilitators

5. What makes it easier for households to access summer meals?
6. What makes it harder for households to access summer meals?

Student Participation and Reach

7. Do you have thoughts about the households who are participating in your summer meals program, as compared to the households who participate in the NSLP only during the school year?
8. Do you have thoughts about the households who do not participate in the summer meals program? Any ideas about reasons why they are not participating?

a. Prompt—For example, distance, lack of awareness, lack of trust, availability of other options, cultural preferences, desire for different menu items than the ones that are available?

Outreach

9. Can you tell me about the communication strategies that are used to inform households about summer meals? When do you usually begin – was that different this year?

a. Prompt—Which strategies do you feel were more successful or less successful?

10. What kinds of family outreach do you do yourselves?
11. What kinds of family outreach do community partners do to help you? We are particularly interested in your experience with organizations that promote the summer meals programs, including End Hunger Connecticut! and UConn Extension’s Put Local on Your Tray program.

Community Partners/Engagement/Augmenting Programs

12. Have there been any community organizations, including the food banks in Connecticut or the local food pantries in your district that you have worked with?
13. Has your district worked with any organizations such as End Hunger Connecticut! or UConn Extension to increase summer meal program participation? What has been your experience working with these organizations?
14. What support could organizations provide to help increase summer meal program participation that they are not already doing?

Long-term Future Needs

15. Are there things you have learned over the years that can help us improve any part of the current summer meal service?
16. What kinds of resources and support would you find helpful?

Wrap-Up

17. Do you have any other thoughts you would like to share with us?
